# Supplementary material for: Are carbon emissions trading and green financial instruments synergistic? -Comprehensive quantitative research based on content analysis
Source: PLoS One. 2024 Mar 7;19(3):e0298601. doi: 10.1371/journal.pone.0298601 (PMC10919601; doi:10.1371/journal.pone.0298601)
Supplement: S1 Data — (ZIP) [file pone.0298601.s001.zip › Data/地级市Z_F工作报告.zip!/╔╜╬≈/│ñ╓╬╩╨/└┤╫╘╣1⁄2╓┌║┼í╛┬φ┐╦╩2╛▌═°í┐_2013.docx]

各位代表：

现在，我代表市人民政府向大会报告工作，请予审议，并请市政协各位委员和其他列席人员提出意见。

一、2012年工作回顾

　　2012年，我市经济社会发展的各项工作取得了新成绩，在全面建成小康社会的征程中迈出了新步伐。（一）经济保持平稳较快发展。全市地区生产总值完成1328.6亿元、增长10.6%，财政总收入302.1亿元、增长18.2%，一般预算收入133.5亿元、增长27.9%，固定资产投资867亿元、增长26.2%，城镇居民人均可支配收入22548.9元、增长12%，农民人均纯收入8120元、增长14.5%，连续三年快于城镇居民收入增幅。重点项目完成投资1433亿元，招商引资实际到位资金590亿元。经济总量继续保持全省领先位次。（二）产业发展得到新的提升。第一产业方面，粮食总产量达到15.9亿公斤，创历史新高。新增设施蔬菜面积10.77万亩，占到全省新增面积的一半。规模以上农业产业化龙头企业达到296家，销售收入突破百亿元，农产品加工转化率达到53%。第二产业方面，煤、焦、冶、电改造提升步伐进一步加快，新兴产业加速发展，完成投资246亿元，增长38.7%，占工业总投资的57.5%，首次超过传统产业投资，新兴产业增加值增幅高于地区生产总值增幅12.2个百分点。第三产业方面，社会消费品零售总额372.6亿元、增长16.1%，进出口总额11.5亿美元、增长47.5%，旅游总收入达到166.5亿元、增长31.9%，第三产业增加值占地区生产总值的比重较上年提高0.5个百分点。（三）先行先试激发新的活力。扎实推进转型综改试点县、试点园区和重大标杆项目建设。大胆探索，先行先试，争取到19个国家级试点和18个省级试点。大力推进行政审批制度改革，市级审批事项由461项精简到116项，精简75%。全面开展城乡建设用地增减挂钩、矿业存量土地整合利用、工矿废弃地复垦试点工作，有效保障了项目用地。建立了多个院士、博士工作站，与中科院、北京大学、天津大学等50多所国内外知名高校和大型科研院所建立了合作关系。长治县华南纸业在天津股权交易所挂牌交易。（四）上党城镇群建设积极推进。按照“一核双圈”的城镇化战略构架，全面实施《上党城镇群行动计划》。大力推进主城区旧城改造，主干道畅通工程和部分城中村改造工程都已启动实施，一批大县城建设的城建重点工程陆续竣工。规划建设了53个重点镇和240个中心村。全市城镇化率达到45.31%，比上年提高1.88个百分点。市域城镇化工作综合考评全省排名第一。（五）生态环境建设得到加强。深入实施绿色生态、“两地”保护和十大节能工程，万元地区生产总值综合能耗下降3.96%，节能工作连续六年排名全省第一，二氧化硫、化学需氧量等六项主要污染物排放量均完成省定任务。完成造林52.9万亩，生态防护功能进一步提高。城市建成区绿化覆盖率达到45%，人均公园绿地面积10.5平方米。市区空气质量二级以上天数357天，稳定达到国家二级标准，在环保部监测的113个重点城市中连续5年名列华北第一。（六）社会建设和民生保障水平稳步提高。全面完成省政府确定的第二轮“五个全覆盖”工程。完善义务教育阶段经费保障机制，为2.4万名普通高中家庭经济困难学生免除学费。新增城镇就业5万人，超额完成省定任务。城镇养老、医疗、失业等社会保险覆盖率保持在95%以上，新型农村合作医疗参合率达到99.5%。在全省率先实施“食品久安工程”。卫生工作在全省综合排名实现 “九连冠”。开工建设各类保障性住房21629套，竣工2598套。实施两大片区扶贫开发和64个整村推进扶贫项目，完成易地搬迁6900人，又有3.9万贫困人口稳定脱贫。继续开展文化低保惠民活动，进一步丰富人民群众文化生活。不断强化安全生产，事故起数和死亡人数分别比上年下降20.6%和0.86%。人口计生工作成效显著，老龄、妇女儿童、残疾人、红十字会、慈善救助等事业健康发展，民族宗教、双拥共建、国防动员、民兵预备役等工作都取得新成绩。新闻出版、体育健身、文物保护、外事侨务、人民防空、防震减灾、气象、统计、档案、保密、地方志等各项事业全面进步。（七）民主法制建设进一步加强。自觉接受人大、政协的监督，认真执行市人大及其常委会的各项决议，大力支持各民主党派、工商联、无党派人士和人民团体参政议政，广泛听取各方面的意见和建议，人大代表建议和政协委员提案办复率达到100%。坚持依法行政，深入开展“六五”普法。党风廉政建设和反腐败工作取得新成效。不断加强社会管理和维稳工作，严厉打击各类严重刑事犯罪活动，深入开展人民调解，及时解决了一批信访突出问题，实现了十八大期间进京“零上访”。积极开展群众性精神文明创建活动，在2012年全国城市文明程度指数测评中，名列全国地级城市第15名，被中央文明委表彰为“全国未成年人思想道德建设工作先进城市”。

各位代表，以上成绩的取得，是省委、省政府和市委正确领导的结果，是市人大、市政协和社会各界有效监督、大力支持的结果，是全市上下群策群力、不断进取的结果。在此，我代表市人民政府，向全市人民，向驻市部队、武警官兵、公安干警，向各民主党派、人民团体，向尽心履职的人大代表、政协委员以及所有关心支持长治发展的社会各界朋友，表示衷心的感谢和崇高的敬意！

在总结成绩的同时，我们应当更加清醒地看到，目前全市经济社会发展中还存在不少需要引起高度重视、认真研究、有效解决的问题：一是一煤独大的倚重型产业结构没有实质性改变，促转型有竞争力的项目不多，经济增长和转型发展都面临很大压力，结构调整、项目建设必须大力推进；二是主城区建设和市域城镇化水平需要加速提升，特别是在缩小城乡差距、区域差距，解决公共资源不均衡、不充分以及完善城市基础设施、加强城市管理等关乎人们现实生活的问题上还要下大功夫；三是安全生产基础不牢，善福煤矿透水事故和天脊苯胺泄漏事故的教训再一次深刻地警示我们，安全生产工作还需抓实再抓实；四是社会民生事业还欠账较多，教育、医疗资源的量与质都还不足、不优，配置也不尽合理，就业、社会保障工作还需加大努力；五是政府自身建设仍有不小差距，少数部门政令不畅、效率低下，吃拿卡要、以权谋私的现象依然存在，整体的发展环境还要改善再改善。对此，我们要下定决心、勇于担当、加力解决。

二、2013年工作安排

　　2013年是全面贯彻落实党的十八大精神、实施“十二五”规划承前启后的关键一年，是推进转型跨越发展、为全面建成小康社会奠定坚实基础的重要一年。今年政府工作的总体要求是：高举中国特色社会主义伟大旗帜，以邓小平理论、“三个代表”重要思想、科学发展观和党的十八大精神为指针，以转型综改试验区建设为统领，以加快转变经济发展方式为主线，以增进人民福祉为根本，紧紧围绕市委十届五次全会确立的实施“五五”战略、率先全面小康的发展目标，保增长、上项目、抓转型、惠民生、强安全、促稳定，打造长治经济社会发展升级版，奋力开创全市转型跨越新局面，为在全省率先全面建成小康社会奠定坚实基础。

今年全市经济社会发展的主要预期目标是：地区生产总值增长11.5%左右，全社会固定资产投资增长25%，社会消费品零售总额增长15.5%，财政总收入和一般预算收入均增长13%，城镇居民人均可支配收入和农民人均纯收入分别增长11%和13%，新增城镇就业4.2万人，城镇登记失业率控制在4.2%以内，居民消费价格总水平涨幅控制在3.5%左右。

约束性指标是：万元地区生产总值综合能耗下降3.5%，万元工业增加值用水量下降5%，万元地区生产总值二氧化碳排放量下降3.7%，二氧化硫、化学需氧量、氨氮、氮氧化物、烟尘、粉尘排放量完成省下达任务。

各位代表，实现以上预期目标任务，决非易事，责任重大。大家知道，一段时期以来，全球经济复苏乏力，国内经济增长动力不足，一季度全国、全省经济增速分别放缓0.2和0.8个百分点。受此影响，主导我市经济增长的主流企业生产经营困难，煤炭、焦炭、化工、冶金等产量下降、价格下跌、效益下滑，加之去年几起安全生产事故发生后，部分煤矿、化工企业停产整顿，以及去年全市经济指标基数较大等原因，因此今年一季度我市主要经济指标增速放缓，在全省位次后移。进入二季度后，虽然煤矿、化工等企业陆续复工复产和一些新上项目竣工投产，全市经济增长出现回升迹象，但距完成阶段性目标任务还有较大差距。各位代表，面对当前的严峻形势，我们必须坚决贯彻落实省委、省政府加快转型跨越发展的总体部署，不辜负省委、省政府对长治提出的要发挥好率先作用的殷切期望，增强忧患意识和进取精神，对标一流、众志成城、奋力赶超；我们必须按照市委十届五次全会确立的实施“五大攻坚”、推进“五大工程”、打造“五大产业集聚区”、走出“五条路子”、建设“五宜”长治的要求，把保增长、上项目、抓转型、惠民生、强安全、促稳定作为今年全市经济社会发展的重要抓手，以科学严谨的态度、敢闯敢冒的胆识、立志大干的精神、求真务实的作风，抓好以下八个方面的工作，力争各项工作在全省“争一保三”。

（一）全力以赴保持经济增长。以降成本、增产量、促销售、占市场为举措，加强对重点产业、重点企业和主要产品的帮扶支持力度，抓好现有企业的正常生产和挖潜增效，着力培育新的经济增长点。加强税源管理，严格税收征管。增强统计工作的科学性、规范性，切实发挥好对经济运行的分析监测预警作用。加快实施“千企百强”培育工程，形成一批销售收入10亿元、50亿元、100亿元以上的“三大企业方阵”，倾力打造引领长治经济发展的旗舰团队。煤炭行业要在保证安全的前提下发挥主力军作用，力争增加值增长14%以上；焦炭行业要加快重组整合步伐，完成增加值增长15%以上；冶金行业要努力提高效益，实现增加值增长20%以上；电力行业要降低生产成本，增加值增长13%以上；先进装备制造、生物医药、特色农产品加工、现代煤化工、新能源、新材料等新兴产业要加大培育发展力度，确保增加值增长30%以上；服务业要在扩大规模、做强做大上狠下功夫，力争增加值增长12%以上。特别是要大力扶持发展民营经济和中小微企业，建设市级中小微企业公共服务网络平台和小微企业创业基地等服务平台，新增千户小微企业和15个销售收入超亿元的“小巨人”企业，力争民营经济增加值占地区生产总值的比重提高2个百分点，使之真正成为带动就业和保增长的生力军。

（二）百折不挠抓好项目建设。今年我市的项目建设任务十分繁重，1至4月份我市项目落地率等几项实质性指标在全省排名靠后。因此，我们不仅要把项目的储备、签约做好，更要把着力点放在项目的落地、开工、建设、投产达效上。现在项目落地难的首要问题是项目的选址80%不在土地利用总体规划建设用地范围内，加之其它一些因素，这就造成项目落地的大大延缓。因此，各级行政部门尤其是市县两级的发改、国土、环保、监察部门必须加紧制定和落实项目的先期预审协调和快速联办联批机制，以及“一站式保姆服务”制和严肃问责制，切实做到想项目所想、急项目所急、办项目应办，高效、规范地推动项目的落地、开工、建设、投产，确保2011年之前开工的项目80%投产达效，2012年开工的项目50%建成，今年新开工的项目50%主体完工，全年重点工程投资完成1420亿元。与此同时，还要下大力气招商、立足优势招商、瞄准产业政策招商、盯住大企业大集团招商，在专家论证、科学评估的基础上，上真转型、真绿色、真富民、真实干的项目。要加强与东部沿海省份、国内外友好城市的合作联系，主动承接发达地区产业转移，争取更多的世界500强和国内著名企业来我市投资发展。今年要力争引进落地1到2个投资百亿元以上的项目，3到5个投资50亿元以上的项目，20个投资10亿元以上的项目，各类产业园区招商引资额要增长40%以上。

（三）综改试验引领转型发展。要抓住先行先试这个灵魂，突出资源型经济转型这个主题，以更大的胆识和气魄推进综改试验区建设，率先闯出一条资源型城市转型发展的路子。按照我市综改试验三年《实施方案》和2013年《行动计划》的要求，今年要重点做好两个方面的工作。

一是推进改革和创新。重点推进行政审批制度、户籍制度、创新“五规合一”规划编制、用地管理、金融创新5项重大改革。进一步减少行政审批项目，尽快启动运行新的行政审批服务大厅，切实做到行政审批“三集中、三到位”。放宽城镇户籍落户条件，允许在城镇常住的农村人口落户城镇，与原住居民享有同等待遇。探索建立经济社会发展、城乡建设、土地利用、产业发展、生态环境保护等规划编制的协调机制，实现各规划无缝对接。创新用地机制，全面推进城乡建设用地增减挂钩，积极推进矿业存量土地整合利用，扩大工矿废弃地复垦利用试点范围，确保耕地占补平衡和总量动态平衡，要切实提高土地投资强度，实现高效集约用地和土地效益最大化。社会发展规律告诉我们，支撑现代经济社会持续发展的根本出路，在于信息化支持下的科技创新和金融创新，这就是创新驱动。推进科技创新，当前就是要加强与国家重点高校、科研院所的合作，建立健全以产学研为主体的产业技术创新联盟，对关键技术组织联合攻关。优化人才培养结构，着力培养创新型、应用型、复合型人才，完善人才引进政策，大力引进科技领军人才特别是创新团队。加快金融改革创新，就是要抓住我市被列为全省促进民间融资规范发展试点市的机遇，引导民间资本更多地投向产业发展领域，加快长治商业银行、农村信用社改制步伐，鼓励新型金融组织创新发展，打造在全省有影响的长治金融商务区，加快构建政府引导、市场主导的政、企、银、融相结合的对接互动平台，及时有效地支持项目建设。继续深入开展整顿规范煤炭经营管理秩序、整治主城区规划建设违法违规行为、打击土地矿产资源违法行为、整治工业商业领域改制企业国有资产流失、整治化工企业违法排污行为的“五大战役”，为推进综改试验区建设创造良好的改革发展环境。

二是推进产业转型升级。把经济下行的压力变为结构调整的动力，“低谷时抓转型、高涨时才有好光景”，用好市场倒逼机制，以延伸抓循环、转型上高端的思路推动产业升级。做好产业发展规划，积极推进信息化和工业化的深度融合，全面提升工业新型化水平。大力实施“长治品牌”战略，打造具有长治特色、在全国有名的产业品牌，今年重点在打造七大新兴产业板块上下功夫。

在现代煤化工循环经济板块方面：以潞安、潞宝、天脊、襄矿为龙头，有效整合现有煤化工园区和企业，在襄垣、潞城、郊区、屯留结合部规划建设总面积200~300平方公里的现代煤化工循环产业集聚区，加快推进百万吨级煤基多联产资源综合利用以及煤制甲醇、煤制烯烃、己内酰胺等项目建设，不断延伸煤电化、煤焦化、煤气化、煤油化等煤基产业链，今年全市传统产业循环率和原材料深加工率要分别再提高2个百分点。

在生物医药板块方面：大力发展生物制药和现代中药产品，加快康宝基因工程疫苗、生物雪莲、振东创新药物GMP生产线等项目建设，延伸血液制品链条，完善中药材种植、加工、研发、销售体系，努力把康宝、振东等企业打造成全国的医药旗舰企业。

在先进装备制造板块方面：以成功集团、清华、淮海、澳瑞特、西门子大型特种电机等企业为龙头，大力引进先进技术、先进工艺、高端产品，积极发展煤矿机械、健身器材、精密加工、节能环保装备、电机电器、汽车制造及零部件等项目，加快推进成功汽车变速箱、易通环能低温发电设备、玉华机械再制造等项目建设。

在新能源新材料板块方面：重点发展大尺寸LED背光源、封装、太阳能电池组件等产品，加强高科华上、潞安太阳能、中池联华等企业的合作，努力打造工业硅—多晶硅—电池片—电池组件—太阳能光伏电站垂直产业链；加快推进电力储能、永磁材料、镁合金、轻钢轻混凝土等新材料项目建设；抓住全省建设三大电力基地的良好机遇，推进江苏协鑫集团“晋电送苏”长治煤电一体化基地项目建设，大力发展生物质能发电、太阳能发电、风力发电、余热发电、“四气”综合利用等新能源项目，力争今年新增150万千瓦的装机容量。积极申报国家新能源示范城市和分布式光伏发电规模化应用示范区，加快建设国家级新能源产品质量检验检测中心。

在现代物流板块方面：利用铁路、公路、机场综合交通优势，重点抓好中南铁路长子编组站物流园、郊区老顶山商贸园、长治县太行山农产品物流园、黎城华驰物流联组园等项目建设，全力打造晋东南地区重要的物流集散地。

在文化旅游板块方面：提升完善总体规划，深度开发山水峡谷、红色教育、神话传说、古建文明、休闲度假等旅游资源，征集旅游宣传口号，打造精品旅游线路，打响“八路军”和“太行山”两个品牌，重点抓好武乡“两园一剧”、壶关太行山大峡谷、平顺虹霓大峡谷、襄垣仙堂山、黎城红山公园、沁源灵空山、长治县荫城古镇等特色景区的升级提档。启动长治游客集散服务中心建设，进一步完善旅游要素配套，提升旅游业综合竞争力。

在特色农产品生产加工板块方面：培育壮大长子方兴、壶关紫团、潞玉种业、金泽玉米、武乡鑫四海、沁州黄小米、潞宝金和生等重点农业产业化龙头企业，建设玉米、小杂粮、干鲜果、食用菌、无公害蔬菜、畜禽养殖等专业化生产基地，不断延伸加工链条，提高加工转化率，努力打造上党特色农副产品生产加工区。

同时要积极发展商贸服务、社区服务和家政服务，着力打造15分钟便民商业圈，加快发展教育培训、医疗保健、社会养老和休闲娱乐等服务业，积极发展网上购物、订单采购、服务外包、信息咨询、产品检测等新兴服务业态，提供多样式的消费选择，创新多样式的消费载体。鼓励引导有实力的工业企业和大型农业产业化企业兴办服务业，引导民间资本进入服务业。构建更加开放的经济体系，加强与国内外的资金、技术、设备双向交流，鼓励我市优势企业走出去拓展经济发展的新空间。积极建设硝酸胺、汽车配件、建材、手工刺绣等外贸转型升级示范基地，不断扩大出口规模。

（四）攻坚克难推动城镇化建设。加大城乡统筹发展力度，深入推进“1+6”和“一核双圈”上党城镇群建设，严格执行城乡建设规划，做好城镇布局设计，形成主城区、大县城、重点镇和中心村一体化发展新格局，全市城镇化率要力争提高2个百分点。

提升主城区城市品质。按照旧城改造与新区建设并举、旧城改造先行的原则，以让人民生活更加舒适便捷为目的，今年实施九大城建工程：一是新建改造14条城市道路，二是改造3座铁路立交桥，三是新建5座市政跨河大桥，四是新建8座过街天桥，五是实施“三河一渠”环城水系二期工程，六是新建北一环城东路互通立交桥，七是建设市区至襄垣快速连接线，八是整治30条背街小巷，九是开工建设新区环湖大道。全力推进长临、黎左、黎霍等高速公路建设项目。加快主城区城中村、城边村改造步伐，大力实施保障性安居工程，新开工建设各类保障性住房20455套，基本建成18000套。积极推进城市综合体的规划建设，突出上党地域文化。抓好全国“智慧城市”建设试点，提升城市建设管理水平。不断完善水、电、气、暖、园林等基础设施，年内完成11万户居民煤层气、天然气置换工作。

提高县城综合承载能力。主城区周边的6个县市要提升规划建设标准，努力建设12万人以上的卫星城。加快“1+6”上党城镇群同城化步伐。坚持“产城融合”发展，对上党城镇群6条快速通道沿线500米范围内的土地实行总控，统筹规划建设一批各具特色的产业园区，以园区聚产业、以产业聚人气、以人气聚市气，努力把“1+6”上党城镇群建成“卫星城模式，同城化服务”的城乡一体化发展范式。外围5个大县城要不断完善基础设施，发展核心产业，壮大县域经济规模，以产业集聚带动人口和要素集聚。重点镇要围绕工矿型、商贸型、农业型、旅游型等不同类型，注重发展特色产业，不断完善基础设施，吸纳周边农民就地转移定居，形成承载万人的规模。中心村要积极创新经济发展路径，切实增强集体经济实力，逐步引入城市社区物业管理模式，为农民提供水、电、路、气、卫生、通信等公共服务。

完善城乡统筹发展新机制。加快城市交通体系、市政公用设施、城乡公共服务等向农村延伸，着力构建城乡一体的教育、卫生、文化、就业培训、社会保障公共服务体系。总结推广长治县振兴新区“就地城镇化”的经验，让农民无论进城务工还是住在乡下都能得到良好的生活保障。

（五）毫不松懈促进“三农”工作。农业、农村、农民问题是全部工作的重中之重，必须切实加大对“三农”的投入，确保支农支出高于财政经常性收入的增长。　大力发展现代农业。实施“235”现代农业示范工程，重点培育20个现代农业示范园区、30个销售收入超亿元的大型农产品加工龙头企业和50个“一村一品”示范村，鼓励社会资本投向现代农业，年内全市龙头企业销售收入增长20%以上，农产品加工转化率达到56%以上。强化农产品质量安全监管，构建农产品质量安全监管溯源系统。加强农业龙头企业科技创新，搞好基层农业科技服务队伍建设。积极探索农村土地流转新机制，发展长子双孢菇、振东中药材等一批现代化种植园区，不断提高农业效益和竞争力。扎实搞好病险水库除险加固和中小河流治理，大力实施农田水利灌溉工程，年内完成农田灌溉面积120万亩。切实提高农业综合生产能力，确保粮食总产量稳定在14亿公斤左右。

多渠道增加农民收入。认真落实各项强农惠农富农政策，继续实施“五大增收工程”，新发展10万亩设施蔬菜、20万亩干果经济林、100个规模养殖场、500个农民专业合作社，转移农村劳动力4万人，努力提高农民经营性收入和工资性收入。大力培育新型职业农民和设施农业技术能手，积极发展以种养大户、家庭农场、农民专业合作社、龙头企业为主的新兴农业经营主体。鼓励农民创业、置业、投资、理财，促进农民转移性和财产性收入持续增长，确保农民收入增幅高于城镇居民。扎实推进新农村建设。抓好380个省级新农村重点村建设，提升11个新农村连片示范区建设水平。以60个市级重点中心村为样板，引领全市新农村建设。扎实做好省里确定的农村“五件实事”，投资1.3亿元改造农村危房9200户，投资2亿元易地搬迁农村特困人口1万人，投资2.6亿元完成2268个行政村街道亮化任务，投资2616万元改扩建96所村级幼儿园，全面实施乡村清洁工程。

加大扶贫攻坚力度。产业扶贫、移民扶贫是今年扶贫攻坚工作的重要内容。要按照市场化运作、项目化推进、产业化经营、工业化管理的方式，组织骨干企业对贫困重点县进行产业扶贫开发，大力实施沁县设施蔬菜、平顺花椒芽菜、武乡和黎城核桃经济林等4个片区产业开发项目，启动平顺、壶关、武乡、沁县、沁源五个贫困县百人以下960个自然村移民搬迁工程。深入开展干部下乡住村、领导干部包村增收活动和机关单位定点扶贫工作，1419个贫困重点村实现干部驻村定点帮扶全覆盖，今年再有4.3万贫困人口稳定脱贫。

（六）齐心协力建设“美丽长治”。建设“美丽长治”必须把生态文明建设放在更加突出的位置，珍惜秀美山川河流，保护绿色生态环境。

切实加强对水资源的保护。长治市的长治久安根本在水。浊漳河是我们的“母亲河”，辛安泉是我们的“生命泉”。我们要树立“护水”、“惜水”意识，善待浊漳河和辛安泉。深入开展浊漳河流域焦化、化工行业整顿治理，对浊漳河流域118户焦化、化工企业进行集中整治，查处、取缔浊漳河沿岸废水排放不达标的污染企业。加快推进大水网工程建设，逐步将境内化工企业用水全部置换为地表水。加强漳泽水库上游和周边污染源治理，促进库区生态环境有效改善。实施辛安泉文王山地垒渗漏段治理工程，保护好辛安泉水质。启动“民生引水工程”，将申村水库作为城市应急备用水源和“三河一渠”补充水源，尽快引水入市。

强力治污减排和节能降耗。严格“两高一资”等传统产业的能耗和环保准入门槛，今年再淘汰85万吨落后产能。大力推进燃煤电厂和水泥行业脱硝、非电行业脱硫及畜禽养殖环保设施建设。加强污水、垃圾无害化处理设施建设和运营管理，加快市污水处理厂二期工程建设。落实新上项目的能评、环评制度。建立细颗粒物（PM

2.5）监测发布体系，开展主城区“蓝天行动”。推行排污权和节能指标交易制度，积极利用清洁发展机制（CDM）。完善减排指标考核体系，严格实行一票否决。继续实施十大节能工程，抓好煤炭、冶金、化工、焦化、电力、建材等六大高耗能行业和105户重点企业的节能工作。加强交通运输、公共机构、居民生活等领域节能工作，积极推进再生资源回收试点，再完成67万平方米既有建筑节能改造。

做好植树造林和生态修复。围绕创建国家森林城市和园林县城全覆盖的目标，加快推进造林绿化工程，加大秋季造林力度，完成营造林40万亩，确保森林覆盖率再提高1个百分点，森林蓄积量增加100万立方米。提升城市绿化品质，主干道要三季有花、四季有绿。做好采煤沉陷区、水土流失等地质灾害治理和生态修复工作，严格控制主城区周边的煤炭开采活动，促进全市生态环境持续改善。

（七）全心全意发展民生事业。一切发展为了人民，一切成果人民共享。各级政府都要把改善民生作为一切工作的出发点和落脚点。

积极扩大就业和鼓励创业。统筹做好农村劳动力转移、城镇就业困难人员和退役军人就业工作，重点做好高校毕业生等青年就业工作，启动青年创新创业园区规划。深入开展创业型城市创建活动，实施创业扶持计划，以创业带动就业。进一步拓宽就业渠道，强化就业政策扶持，完善公共就业服务体系，大力发展劳动密集型产业。今年新增城镇就业4.2万人。

全面发展教育事业。进一步加大教育投入，确保全市教育经费支出按法定要求增长。全力办好学前教育，改扩建18所公办标准化幼儿园，在主城区新建3所市直公立幼儿园，缓解城市“入园难”问题。均衡发展义务教育，全面完成义务教育薄弱学校改造，积极推进中小学生向乡镇、县城集中。普及高中阶段教育，今年高中阶段毛入学率达到94%以上。大力发展现代职业教育，完成长治市高级技工学校、太行技工学校整合，按我市产业发展趋势和企业需求设置职业技术教育专业，为长治经济发展培养更多的实用人才。推进科教园区建设，加快长治学院、长治医学院、长治职业技术学院等6所院校新校区的建设。

大力发展医疗卫生事业。加快医药卫生体制改革，开展市二院城市公立医院改革试点，扩大县级公立医院改革试点，同步推进县域医药卫生一体化综合改革，在试点县全部实行药品零差率销售，推动基本药物制度向非政府办基层医疗机构延伸。加快市人民医院的扩建达标升级工作，实施市人民医院二期改造工程。巩固完善县乡村三级卫生服务网络和城市社区卫生服务体系，逐步实现基本公共卫生服务均等化。进一步加强全科医生培养和乡村医生队伍建设，不断提高基层医疗服务水平。深入推进“好娃娃”工程，全面实施免费孕前优生健康检查，扎实开展“长治健苗”出生缺陷干预救助工程，切实提高出生人口素质，稳定低生育水平，促进人口长期均衡发展。

健全覆盖城乡的社会保障体系。城乡居民基础养老金在全省提高10元的基础上，我市再提高5元、达到70元，企业退休人员基本养老金再提高10%。城镇居民医保和新农合财政补助标准再提高40元，达到280元，其中14.5万农村低保户和五保户的个人缴纳部分全部由市级财政负担。城乡低保保障标准每人每月分别提高30元、24元。健全被征地农民社会保障制度，逐步将城市中稳定就业的农民工全部纳入社会保险范围。　 加强和创新社会管理。进一步引深“法治长治”、“平安长治”创建活动。发挥好市委书记市长公开电话办理中心、新建信访接待大厅的作用。整合各类资源，形成全市集中统一的社会网格管理服务平台和信息平台。完善社会治安防控和公共安全体系，做好对特殊人群的管控，严厉打击各类严重刑事犯罪活动。加强“扫黄打非”，搞好文化市场管理。切实强化应急管理和网络舆情工作，完善各种公共危机处理机制，加强应急演练，有效应对公共突发事件。依法管理民族宗教事务，促进社会和谐稳定。把食品药品安全监管作为重大民生问题，加快推进食品药品监管机构改革，完善市县乡村四级网格化监管体系，深入实施“食品久安工程”，重拳打击食品药品违法违规行为，确保人民群众饮食安全、用药安全。认真实施“六五”普法。抓好国防教育、人民防空、民兵预备役工作，完善国防动员体系，深化双拥共建活动。深入开展全国全民健身示范市创建活动，不断提升竞技体育水平。进一步加强审计、对台、气象、档案、保密、外事侨务、防震减灾、地方志等工作。充分发挥工会、共青团、妇联等人民团体的桥梁和纽带作用。关心支持妇女儿童、残疾人、老龄、红十字会、慈善救助等事业。

各位代表，安全生产重于泰山。要树立“大安全”的观念，扎实抓好煤矿、非煤矿山、危险化学品、尾矿库、道路交通、森林防火、特种设备、建筑施工、中小学校等重点行业领域的安全整治。要重监管，严格落实企业主体责任和政府监管责任，坚决执行安全生产法律法规和各项行之有效的制度措施，各种安全生产指令必须做到令行禁止。强队伍，对全市所有安全监管执法人员进行严格培训、科学管理；对所有高危企业一线操作人员、特殊岗位人员进行基本安全知识和技能培训，提高企业职工的安全素质。除隐患，深入开展安全隐患排查治理，建立完善企业安全隐患排查治理体系，实现隐患排查制度化、责任化，从源头上消除各类安全隐患。严追究，严格执行安全生产 “一票否决制”，坚决做到事故处理 “四不放过”，对安全生产违法行为发现一起、查处一起，对责任人要依规按上限从重处罚，以强有力的举措坚决杜绝重特大事故，遏制较大事故，减少一般事故，彻底扭转安全生产的被动局面，确保人民群众生命财产安全！

（八）文化强市构建城市文明。长治不仅有丰富的地下资源，更有深厚的文化底蕴，我们要充分挖掘文化宝藏，让多姿多彩的上党文化造福人民。

促进文化事业大发展大繁荣。扎实推进国家公共文化服务体系示范区建设，提高公共文化服务能力，继续抓好县级文化馆、图书馆建设，提升乡镇（社区）综合文化站和村文体活动室服务功能，积极开展农村电影放映和送书送戏下乡等惠民工程，丰富群众精神文化生活。加大物质文化遗产保护，特别是要对潞安府衙、观音堂等主城区六大古建筑和老顶山进行控制性保护，在做好规划的基础上进行整体修复建设，恢复城市文化记忆，重塑上党古城文化之魂。加强非物质文化遗产传承。鼓励文化精品创作，打造一批精品剧目。实施重大文化产业项目带动战略，大力发展新兴文化业态，培育具有长治特色的现代文化产业体系，打造红色抗战、历史民俗和生态养生三大文化品牌，推动文化旅游、文化创意、文化娱乐、体育健身等产业集聚发展。

传承长治传统美德。践行社会主义核心价值观，大力弘扬太行精神、纪兰精神，切实加强社会公德、职业道德、家庭美德、个人品德和未成年人思想道德建设。引深精神文明创建活动，进一步巩固“三项治理”成果，认真做好全国文明城市迎检工作，努力提高市民的文明素质和城市的文明品位。

各位代表，建设人民满意的人民政府至关重要。各级政府要切实加强自身建设：一要务实节俭廉洁勤政。工作要上去、干部要下去。按照一月要有一旬下基层的要求，深入群众，察实情、知实际，为基层着想、替百姓分忧，敢作为、敢担当，遇到矛盾不回避，碰到困难不退缩，切实解决实际问题。要严格执行中央改进作风的“八项规定”和国务院的“约法三章”以及省市委的各项要求，厉行节约，要“过紧日子”，花好财政的每一分钱。各级干部要深知，权力是人民赋予的，责任是人民重托的，决不能假公济私、公权私用，决不能不给好处不办事、给了好处乱办事，决不能借工程招投标、土地征用、政府采购等为个人徇私情、捞好处，一经发现要严肃惩处，决不宽容。每个人都要尽心竭力履行好职责，勤勤恳恳为人民服务，都要为创造长治良好的发展环境做贡献，都要为树立政府的良好形象添光彩。二要依法民主高效行政。严格按照法定权限和程序履行职责，主动向人大报告工作和向政协通报情况，支持法院、检察院依法独立履行职责，诚恳听取各民主党派、工商联、无党派人士和各人民团体的意见。切实转变政府职能，规范政府行政行为，大力推进政务公开。加强制度建设，完善科学民主决策机制，该集体决策的不能个人独断，应科学论证的不能个人拍板。言必信、行必果，一旦做出决定决策，就要坚决贯彻、严格执行。加强行政效能建设，不说不能办、只说怎么办，首办负责、限时办结，雷厉风行、高效快捷，以一流的效率创一流的业绩。

各位代表，长风破浪会有时，直挂云帆济沧海。让我们紧密地团结在以习近平同志为总书记的党中央周围，在省委、省政府和市委的坚强领导下，凝聚全市人民的智慧和力量，以实施“五五”战略、率先全面小康为目标，万众一心、开拓奋进，共同谱写上党人民美好生活新篇章！
